# A new model mimicking persistent HBV e antigen-negative infection using covalently closed circular DNA in immunocompetent mice

**DOI:** 10.1371/journal.pone.0175992

**Published:** 2017-04-20

**Authors:** Lei Wang, Min Cao, Qing Lu Wei, Zhong Hua Zhao, Qin Xiang, Hui Juan Wang, Hua Tang Zhang, Guo Qi Lai

**Affiliations:** 1 Chongqing Medical University Laboratory Animal Center, Chongqing, China; 2 Chongqing Academy of Science and Technology, Chongqing, China; Indiana University, UNITED STATES

## Abstract

Despite the availability of an effective vaccine, hepatitis B virus (HBV) infection remains a major health problem. HBV e antigen (HBeAg)-negative strains have become prevalent. Previously, no animal model mimicked the clinical course of HBeAg-negative HBV infection. To establish an HBeAg-negative HBV infection model, the 3.2-kb full-length genome of HBeAg-negative HBV was cloned from a clinical sample and then circularized to form covalently closed circular (cccDNA). The resulting cccDNA was introduced into the liver of C57BL/6J mice through hydrodynamic injection. Persistence of the HBeAg-negative infection was monitored at predetermined time points using HBV-specific markers including HBV surface antigen (HBsAg), HBeAg, and HBV core antigen (HBcAg) as well as DNA copies. Throughout the study, pAAV-HBV1.2 was used as a control. In mice injected with HBeAg-negative cccDNA, the HBV infection rate was 100% at the initial stage. HBsAg levels increased up to 1 week, at which point levels peaked and dropped quickly thereafter. In 60% of injected mice, HBsAg and HBcAg persisted for more than 10 weeks. High numbers of HBV DNA copies were detected in the serum and liver. Moreover, cccDNA persisted in the liver tissue of HBeAg-negative mice. In contrast to the pAAV-HBV 1.2 injected mice, no HBeAg was found in mice injected with HBeAg-negative HBV throughout the study period. These results demonstrate the first successful establishment of a model of HBeAg-negative HBV-persistent infection in immunocompetent mice. Compared to pAAV-HBV1.2-injected mice, the infection persistence and levels of serum virological and biochemical markers were approximately equal in the model mice. This model will be useful for mechanistic studies on HBeAg-negative HBV infection and will facilitate the evaluation of new antiviral drugs.

## Introduction

Viral hepatitis type B is a major global health problem caused by the hepatitis B virus (HBV). HBV infection can lead to acute and chronic hepatitis, which may progress to liver cirrhosis and hepatocellular carcinoma (HCC) [1–3]. In a recent report, it was estimated that there are approximately 248 million individuals infected chronically with HBV worldwide [4]. Chronic hepatitis B (CHB) results in more than 600,000 deaths annually from complications of end-stage liver disease and HCC. The serologic pattern in patients with CHB is complicated; two of the most common pathogen-associated states for HBV are hepatitis B e antigen (HBeAg)-positive and HBeAg-negative [5]. In recent years, the prevalence of HBV infection has decreased. At the same time, the spectrum of this infection has changed. Thirty years ago, the wild-type HBeAg-positive virus was the primary epidemic strain; however, the prevalence of HBeAg-negative strains is increasing globally [6–8].

Among the two types of CHB, there are major differences in terms of epidemiology, pathogenesis, natural clinical course, prognosis, and treatment [9–11]. Compared to HBeAg-positive CHB patients, HBeAg-negative patients exhibit low sustained response rates to antiviral therapy, resulting in increased treatment times. Moreover, HBeAg-negative CHB has higher potential for developing into HCC [12]. Therefore, long-term follow-up studies are needed to better understand the natural history and prognosis of HBeAg-negative CHB patients. However, studies on HBV infections, treatment of CHB, and antiviral drug screening still rely on an HBeAg-positive animal model. Until recently, there has been no incentive for creating an HBeAg-negative animal model. This has limited studies on HBeAg-negative HBV infections in terms of mechanisms, treatments, and drug screening. Therefore, an animal model of HBeAg-negative persistent HBV infection is urgently needed.

In the current study, we cloned the HBeAg-negative HBV genome and circularized it into covalently closed circular DNA (cccDNA). We then established an animal model of HBeAg-negative HBV through hydrodynamic injection into the tail vein of C57BL/6J mice [13]. In this model, viral markers persisted for at least 10 weeks.

## Materials and methods

### Analysis of blood samples

Blood samples were drawn by venipuncture from 53 patients with HBV-positive infections at The Second Affiliated Hospital of Chongqing Medical University (Chongqing, China). HBV surface antigen (HBsAg) and HBeAg were detected using an enzyme linked immunosorbent assay (ELISA) at the hospital. The HBV genotype was analyzed by SNaPshot. Probe sequences and reaction conditions were as reported previously [14]. All procedures were in compliance with the Declaration of Helsinki. The study protocol was approved by the Ethics Committee of the Chongqing Medical University.

### Cloning of full-length DNA from an HBeAg-negative viral strain

Full-length HBV DNA was acquired from patient number 11061008, who was diagnosed with a genotype B, HBeAg-negative infection, by the polymerase chain reaction (PCR). Primers for fragment and full-length amplification containing BspQI restriction enzyme sites were designed. The forward primer was 5′-TTATGCTCTTCTTTTTCACCTCTGCCTARTCATC-3′ and the reverse primer was 5′-TCATGCTCTTCAAAAAGTTGCATGGTGCTGGTG-3′. These primers were synthesized by Invitrogen Bio-Tech (Shanghai, China). Subsequently, full-length DNA from the HBeAg-negative virus was cloned into the pEASY-Blunt Simple Cloning vector (TransGen Biotech, Beijing, China). The resulting pEASY-HBV/HBeAg-negative plasmid was sequenced by Invitrogen. The sequencing result was subjected to a BLAST search at PubMed.

### Production of circularized HBV DNA

Circularized DNA was produced by the conventional plasmid-enzymatic ligation method [15–17]. Briefly, the pEASY-HBV/HBeAg-negative plasmid was digested with the BspQI restriction enzyme (New England Biolabs, Worcester, MA, USA). A 3.2-kb linear target fragment was retrieved after agarose gel electrophoresis. Linear HBV was circularized by T4 DNA ligase (New England Biolabs) and then purified using a TIANquick Midi Purification Kit (Tiangen Bio-Tech, Beijing, China).

### Establishment of the animal model

Male C57BL/6J mice (6–8 weeks of age, 18–24 g, specific pathogen-free) were provided by the Laboratory Animal Center of Chongqing Medical University (SCXK (YU) 2012–0001). The mice were maintained under optimal conditions for hygiene, temperature, and photoperiods (12:12 h light: dark), and allowed food and water ad libitum, according to the institutional guidelines for the care and use of laboratory animals.

Thirty-six C57BL/6J male mice were divided randomly into three groups. The experimental group (n = 15) was injected with circularized HBeAg-negative HBV DNA using the hydrodynamic method (2 μg/2 mL/mouse, within 5–8 s) [13]. The control group (n = 15) was injected with pAAV/HBV1.2 (5 μg/2 mL/mouse). pAAV/HBV1.2 was kindly provided by Prof. PJ Chen (Graduate Institute of Clinical Medicine, College of Medicine, National Taiwan University). The blank group (n = 6) was injected with normal saline (2 mL). Serum samples were collected via the tail vein at 1 and 3 days, and at 1, 2, 3, 4, 5, 6, 7, 8, 9, and 10 weeks after injection. After 3 and 10 weeks, three mice from each group were sacrificed by cervical dislocation after intraperitoneal injection of 10% chloral hydrate, and serum and liver tissue were collected. All animal procedures were approved by the Ethics Committee of Chongqing Medical University (permit number: 2013039). All surgical procedures were performed using anesthesia, and all possible efforts were made to minimize animal suffering.

### Detection of serum HBV-specific markers in mice

Mouse sera were tested for HBV-specific markers (HBsAg, HBeAg, and viral DNA) and alanine aminotransferase (ALT) at distinct time points. The levels of HBsAg and HBeAg in serum were detected using a radioimmunoassay diagnostic kit (Beijing North Biotechnology Research Institute, Beijing, China). Serum levels of ALT were detected using a mouse ELISA kit (Wuhan Colorful Gene Biological Technology, Wuhan, China) according to the manufacturer’s instructions. HBV DNA was detected in serum by real-time fluorescent quantitative PCR (qPCR). DNA was extracted from serum (50 μL) using the TIANamp Virus DNA/RNA Kit (Tiangen Bio-Tech), and 2 μL of total DNA was used for qPCR. The HBV copy number was quantified using SYBR Green assays with FastStart Universal SYBR Green Master mix (Roche Diagnostics, Mannheim, Germany). Primers for amplification of HBV DNA fragments were designed according to the conserved region of the HBV gene. The sequences were as follows: forward primer (F402), 5′-CCTCTTCATCCTGCTGCT-3′; reverse primer (R718), 5′-AACTGAAAGCCAAACAGTG-3′. The standard curve was established using pEASY-HBV/HBeAg-negative HBV DNA at known titers of 5 × 10^3^, 5 × 10^4^, 5 × 10^5^, 5 × 10^6^, 5 × 10^7^, and 5× 10^8^ copies/μL. The PCR reaction was performed with the following conditions: 3 min at 95°C, followed by 40 cycles of 20 s at 94°C, 30 s at 50°C (qPCR/melt data acquisition), and 30 s at 72°C.

### Detection of viral titers and cccDNA in mouse liver tissue

HBV DNA was quantitated in liver tissue by qPCR as described previously. cccDNA was detected by rolling circle amplification (RCA)-PCR. Briefly, viral DNA from mouse livers at 21 and 70 days post-injection (dpi) was treated with plasmid-safe ATP-dependent DNase (PSAD) (Epicentre, Madison, WI, USA) for 12 h at 37°C. Subsequently, RCA was performed with primers and reaction conditions in accordance with the literature [18]. The step 1 primer combination was as follows: 1 μL of DNA was added to eight primer mixtures with a volume of 2 μL and 10× phi29 buffer to a final volume of 10 μL. The DNA mixture was denatured at 95°C for 3 min and then cooled to room temperature in stages (50°C for 15 s, 30°C for 15 s, and 20°C for 10 min) before being placed on ice. Step 2 amplification was performed as follows: sample mixtures were combined with 10 μL of reaction mixture containing 10× Phi29 buffer, 3 μL of dNTPs, and 10 U of Phi29 DNA polymerase (New England Biolabs). Reactions were performed at 30°C for 16 h and terminated at 65°C for 10 min. Finally, the production of RCA was identified by PCR using a pair of cccDNA-selective labeled primers that target the gap region between the two direct repeat regions of the viral genome [19,20].

### Hepatic histopathological and immunohistochemistry (IHC) analyses

Hepatic histopathologic changes were observed by hematoxylin and eosin (HE) staining. The location and expression of viral antigens in liver tissue were detected by IHC staining [21]. Paraformaldehyde-fixed paraffin-embedded tissue sections (4.5-μm thickness) were stained using HE and IHC. HBsAg and HBV core antigen (HBcAg) expression was determined in the liver sections by IHC using horse anti-HBsAg (1:1000) (ab9193, Abcam, Cambridge, UK) and rabbit anti-HBcAg (1:1000) (B0586, DAKO, Glostrup, Denmark). For the negative staining control, specimens were treated with normal saline only.

### Statistical analysis

Data are expressed as the mean ± standard error of the mean (SEM) of at least three independent experiments. Statistical analysis was performed using the independent-samples t-test. P<0.05 was considered significant.

## Results

### HBV analysis

We surveyed clinical samples from 53 patients. Data regarding HBV genotypes, as well as HBsAg and HBeAg contents, were gathered. Twenty-seven (50.9%) of the HBV-infected patients were HBeAg-negative. Among the 53 patients, one patient presented the A genotype, whereas 38 were infected with hepatitis B, and 14 had the C type. The proportion of male patients was 92%.

### Cloning of an HBeAg-negative viral strain

**Fig 1:**
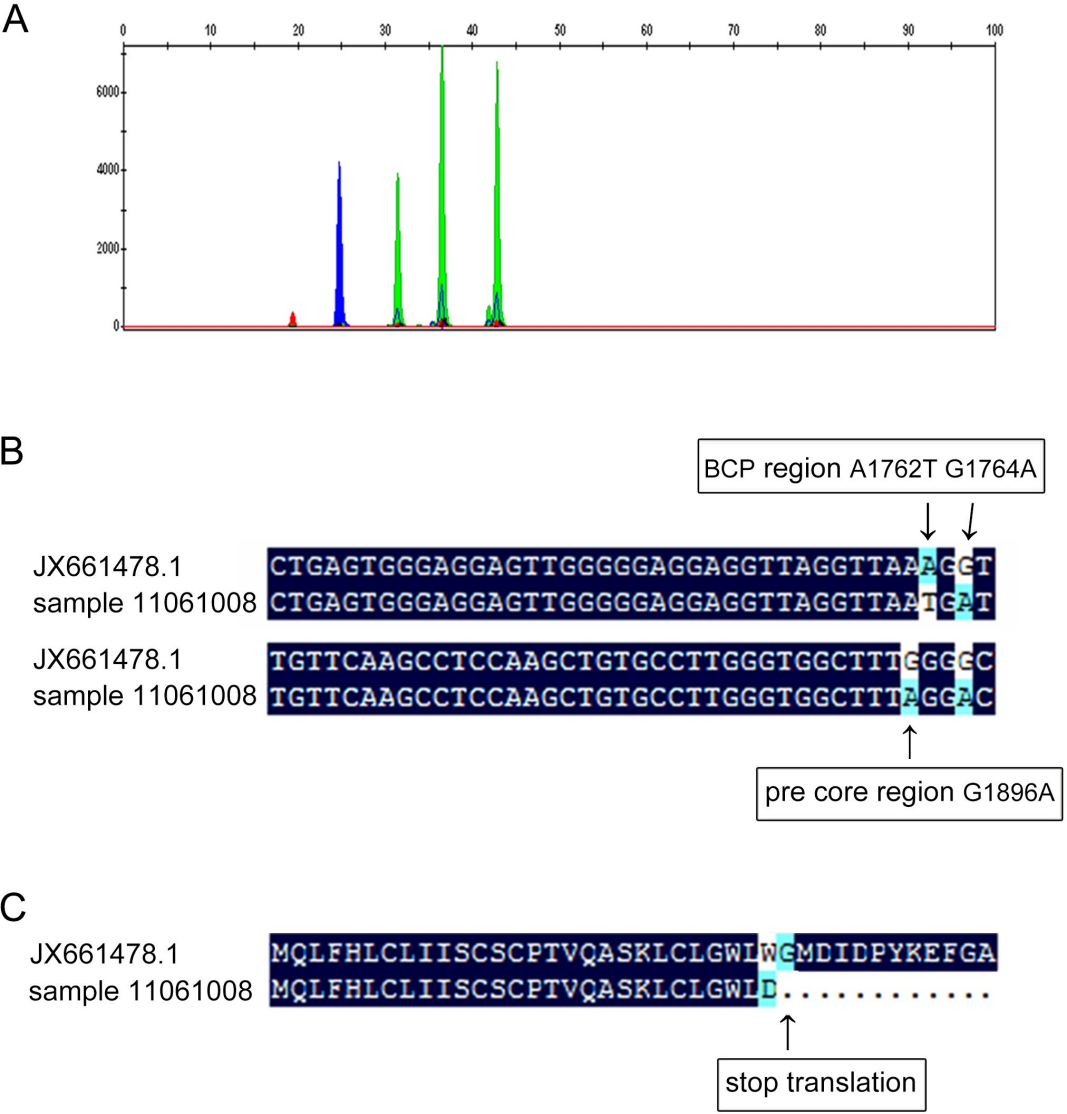
Identification of the viral strain. (A) Results of genotype identification: red, green, blue, and black peaks indicate the bases T, A, G, and C, respectively. (B) The BLAST sequence search using PubMed. JX661478.1 is from GenBank. Sample 11061008 is the sequence of the pEASY-HBV/HBeAg-negative plasmid. (C) Amino acid sequence of the protein BLAST. JX661478.1 is the partial sequence of the e protein from GenBank. Sample 11061008 is the partial sequence of the e protein in the pEASY-HBV/HBeAg-negative plasmid.

The viral genotype from the one blood sample (no. 11061008) that we selected is shown in Fig 1A. The results indicated HBV A-type for ACAA, B-type for GAAA, C-type for GCCA, and D-type for GCAT. The peak signal was obtained with GAAA. Therefore, the HBV genotype was B. The pEASY-HBV/HBeAg plasmid DNA sequence was subjected to a BLAST search using the NCBI database, which showed that the sequence was 98% identical to GenBank sequence JX661478.1. The four overlapping open reading frames were complete.

We next analyzed the P, S, X, and C regions by nucleotide and protein BLAST. The mutation rate was insignificant except for G1896A, G1899A, A1762T, and T1764G point mutations in the C region [6] (Fig 1B). The presence of basal core promoter mutations in combination with the G1896A and G1899A mutations is often seen in fulminant hepatitis [22]. G1896A could create a TAG stop codon at codon 28 of the pre-core (preC) protein and abolish HBeAg expression at the translational level (Fig 1C). However, 1899 is downstream of 1896, and hence is not translated into amino acids. We had just established a HBeAg-negative mouse model and therefore did not consider it. Subsequently, HBeAg-negative cccDNA was generated through plasmid-enzymatic ligation (S1 Fig).

We have verified the cccDNA by agarose gel electrophoresis before hydrodynamic injection (S2 Fig). Lanes 3 and 7 show products after ligation and ligated products + PSAD, respectively. PSAD was used to digest relaxed circular HBV DNA, and double-stranded DNA and single-stranded DNA. Because HBV DNA contains EcoRI restriction sites, linear HBV DNA could become two fragments and cccDNA could become a 3.2-kb fragment following digestion with EcoRI.

### Persistence of virus-specific serum markers in mice

**Fig 2:**
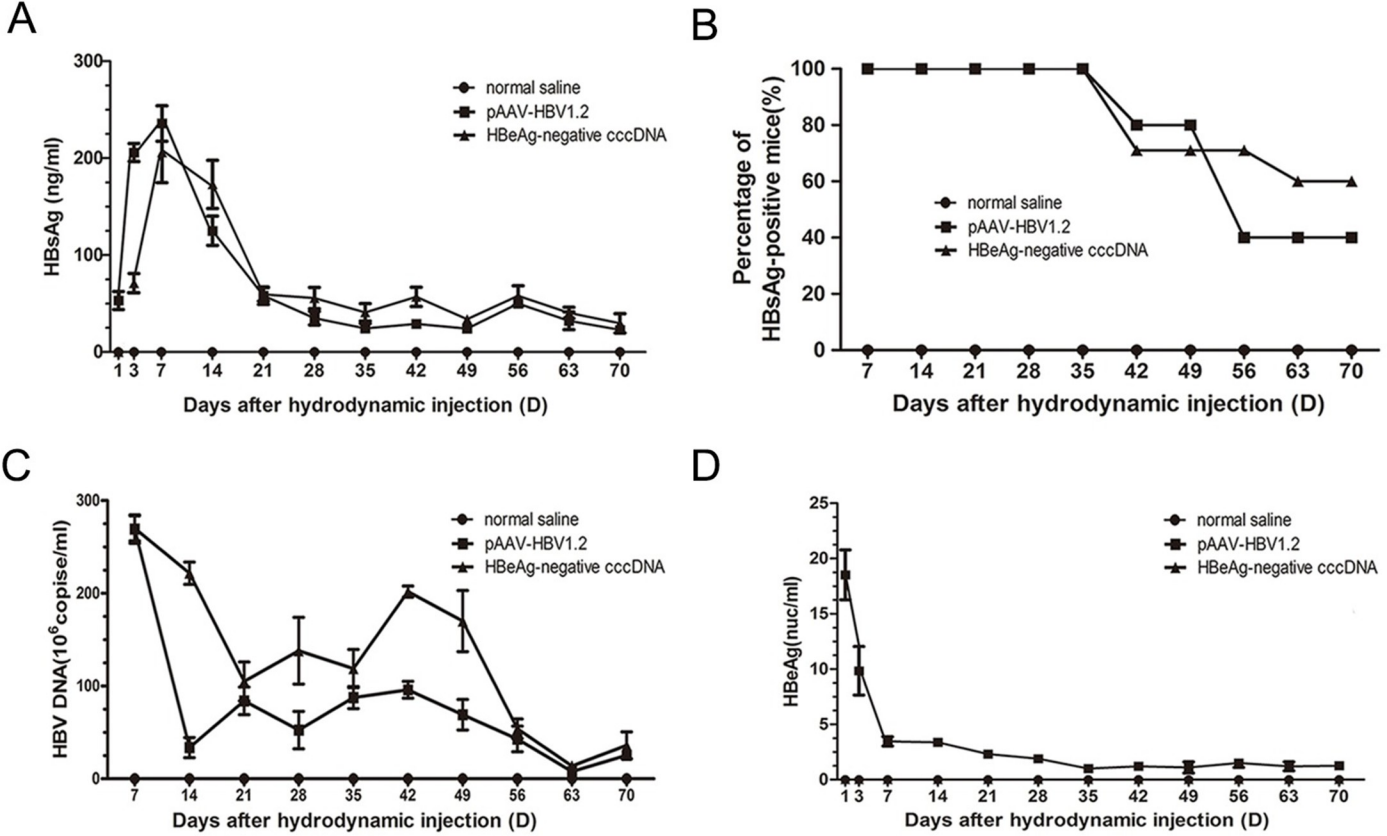
Persistence of virus-specific markers in mouse serum. (A) Levels of HBsAg in sera after hydrodynamic injection. The detection limit was 15 ng/mL. (B) Positivity of HBsAg in serum at different time points after injection (control and experiment group n = 15, blank group n = 6). (C) Levels of HBV DNA in sera. (D) Levels of HBeAg in sera after injection. The detection limit was 1 ng/mL.

Levels of HBsAg and HBeAg in mouse serum were detected at predetermined time points by radioimmunoassay (Fig 2). HBsAg could be detected at 1 and 3 dpi in the control and experimental groups, respectively; these levels increased up to 1 week, at which point they reached their peak and dropped quickly thereafter in both groups. The blank group remained negative throughout (Fig 2A). Sixty percent of experimental mice remained HBsAg-positive at 70 dpi (Fig 2B).

Serum HBV DNA levels from all mice, except the blank group, peaked during the first week (Fig 2C). The concentrations of HBV DNA averaged 2.69 × 10^8^ and 2.70 × 10^8^ copies/mL of sera in the control and experimental groups, respectively. Thereafter, the viral loads decreased and were undetectable in some serum samples at later time points; however, they remained high in the serum samples from the HBsAg-positive carrier mice. The average viral DNA concentration in serum samples from high-titer HBsAg-positive mice at 70 dpi was 3.6 × 10^7^ copies/mL in the experimental group. As expected, HBeAg could only be detected in the control group when mice were HBsAg-positive and had high levels of HBV DNA (Fig 2D). The level of HBeAg was high at 1 dpi but declined rapidly thereafter. At all time points tested, all other groups were HBeAg-negative. Forty percent of C57BL/6J mice expressed HBsAg and HBeAg for at least 10 weeks in the control groups (Fig 2B).

### Persistence of HBV and cccDNA in mouse liver

**Fig 3:**
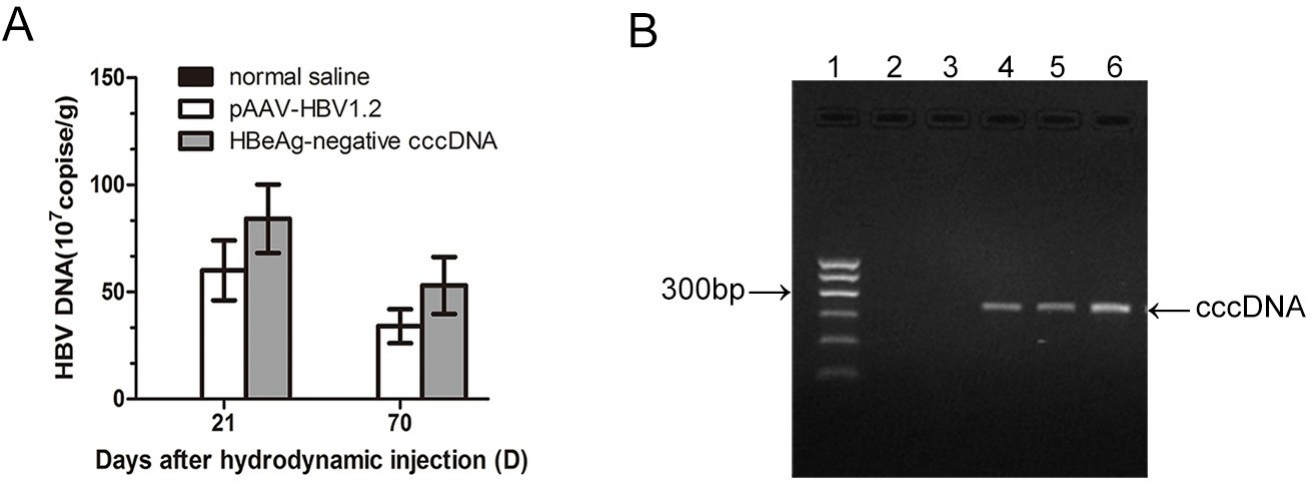
Detection of viral titers and cccDNA in mouse liver tissue. A. HBV DNA was detected in liver by qPCR. B. Detection of cccDNA by rolling circle amplification-PCR. Lane 1: 100 bp ladder; lane 2: pEASY-HBV/HBeAg-negative plasmid as the template; lane 3: linear HBV as the template. The pEASY-HBV/HBeAg-negative plasmid was digested by BspQI and the products served as a template; lane 4: experimental group at 21 days post-injection (dpi); lane 5: experimental group at 70 dpi; lane 6: circularized DNA served as the template.

HBV persisted at greater than 10^8^ copies/mL in livers of control and experimental mice at 21 and 70 dpi (Fig 3A). The level of HBV DNA in the liver was much higher than that in the sera at the same time points. cccDNA could be detected by RCA-PCR in liver tissue at 21 and 70 dpi in the experimental group.

PSAD + RCA + PCR increases the sensitivity and specificity of HBV cccDNA detection compared to Southern blotting [19]. The products were separated by 2% agarose gel electrophoresis and visualized under UV light (Fig 3B). Agarose gel electrophoresis was negative for the pEASY-HBV/HBeAg-negative plasmid and linear HBV templates (lanes 2 and 3). cccDNA, as the positive control, was amplified and identified by PSAD + RCA + PCR. The sensitivity of this method was found to be one copy of cccDNA (S3 Fig). This technique resulted in a 332-bp objective strap that was the cccDNA (lane 6). cccDNA was detected at 21 and 70 dpi (Fig 3B, lanes 4 and 5) in the experimental group.

### Expression of HBcAg and HBsAg in mouse liver

**Fig 4:**
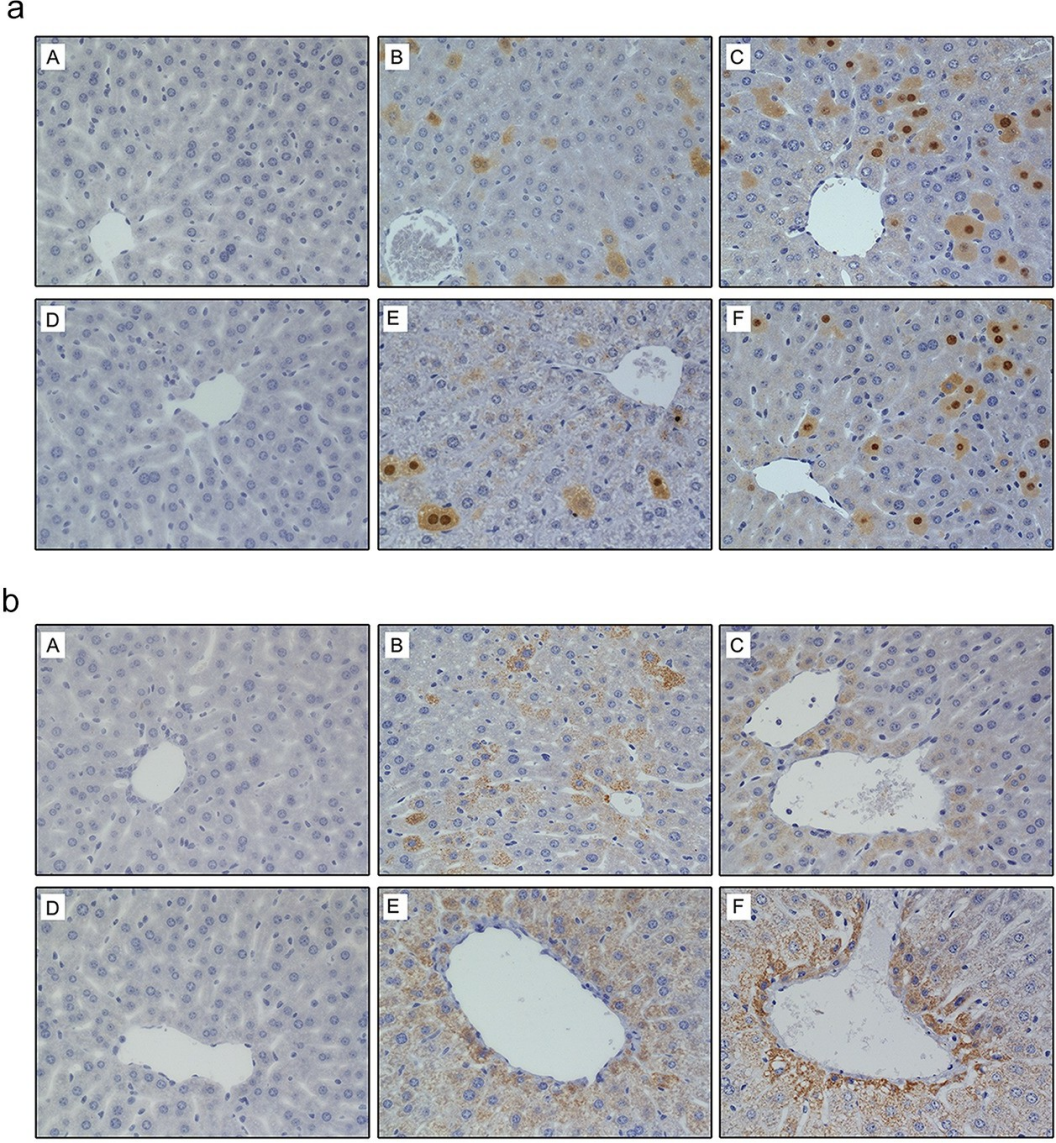
Expression of HBcAg and HBsAg in hepatic tissue of mice at 21 and 70 days post injection (dpi) determined by immunohistochemistry (IHC). (A) IHC for the expression of HBcAg. (B) IHC for the expression of HBsAg. IHC in liver tissue of 21 dpi animals injected with (a) normal saline, (b) pAAV/HBV1.2, and (c) circularized HBV DNA. IHC in liver tissue of 70 dpi animals injected with (d) normal saline, (e) pAAV/HBV1.2, and (f) circularized HBV DNA. (Original magnification: ×400).

IHC staining of the liver 3 and 10 weeks after hydrodynamic injection resulted in a heterogeneous pattern of HBcAg-positive hepatocytes, identified as yellowish-brown granules, except for in the blank group. HBcAg-positive hepatic cells (both nuclear and cytoplasmic) were interspersed randomly throughout the hepatic lobule and tended to localize to the central lobule (Fig 4A). HBsAg was detected using the same method. HBsAg expression was hepatocellular and cytoplasmic, and tended to localize to the central lobule, except for in the blank group (Fig 4B).

### Histopathological changes in the liver

**Fig 5:**
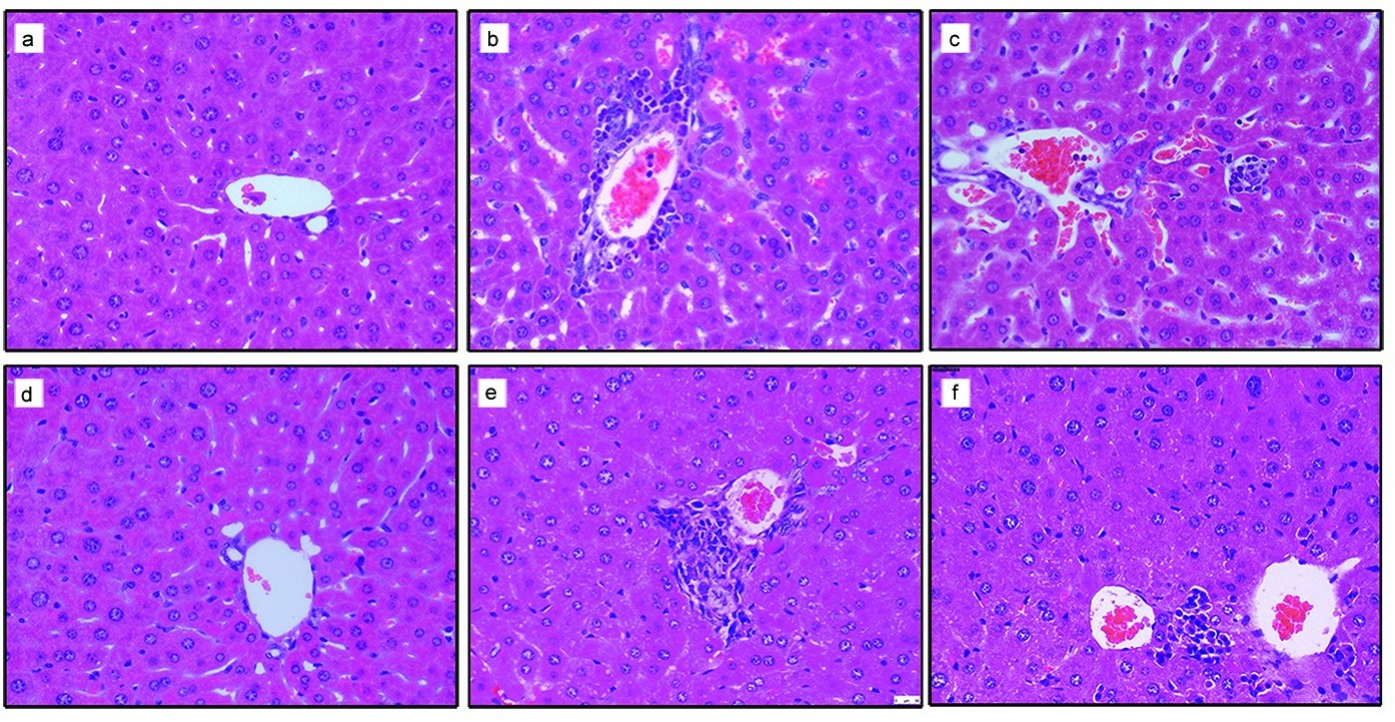
Histopathological changes in mouse hepatic tissue at 21 and 70 dpi assessed by hematoxylin eosin (HE) staining. HE assay for hepatic histopathological changes of 21 dpi animals injected with (a) normal saline, (b) pAAV/HBV1.2, and (c) circularized HBV DNA. HE assay for hepatic histopathological changes at 70 dpi in animals injected with (d) normal saline, (e) pAAV/HBV1.2, and (f) circularized HBV DNA. (Original magnification: ×400).

The livers of C57BL/6J mice at 21 and 70 dpi were stained with HE (Fig 5). In contrast to the histology findings and positive biochemistry for viral biomarkers, long-term expression of HBV in these carrier mice did not lead to serious liver damage. These mice had normal levels of ALT (data not shown) confirming this finding. All livers at 21 and 70 dpi had multiple foci of mononuclear cell infiltration, except for the blank group.

## Discussion

Animal models of human disease serve as important tools to study pathogenic mechanisms and evaluate drugs before clinical treatment. There are many animal models of HBV infection. Mouse models have significantly aided our comprehension of the HBV lifecycle and pathogenesis [23]. However, they have also been focused solely on HBeAg-positive infection; there are few animal models of HBeAg-negative infection.

In the current study, we surveyed a small number of clinical samples from HBV-positive patients. The results showed that 50.9% were HBeAg-negative, and the B type comprised 67.9% of cases. The proportion of male patients was 92%, which was consistent with recent reports [6–8]. Based on clinical data, we used a serum sample from a male patient diagnosed with CHB, with HBeAg-negative and HBV B type virus, as the template. Circularized HBV DNA was created based on the plasmid-enzymatic ligation method. HBeAg-negative cccDNA was injected into C57BL/6J male mice via the hydrodynamic method. In doing this, an HBeAg-negative HBV mouse model was established successfully. The HBeAg-negative infection persisted and HBV markers, including HBV DNA, HBsAg, HBcAg, and cccDNA, were detected at up to 10 weeks using this model. Serum ALT levels were normal and inflammation in the liver was minimal. Throughout the study, we used pAAV-HBV1.2, an HBeAg-positive and widely used viral construct, as the control. The purpose of using pAAV-HBV1.2 was to determine whether HBeAg can be expressed in this mouse. Compared to the control group, the persistence of viral infection and the levels of serum virological and biochemical markers were approximately equal.

We suggest that our model simulates HBeAg-negative infections in CHB patients because these patients also have normal ALT levels and HBV DNA loads above 2000 IU/mL [24]. In a recent report, HBeAg-mediated inhibition of HBV replication and transcription was observed in cell culture models [25]. In addition, there have been recent reports that down-regulation of an intrahepatic host gene, which could participate in the innate immunity pathway, was more pronounced in HBeAg-negative patients, and some intrahepatic host genes were significantly less repressed in HBeAg-positive patients [26]. This suggested that HBeAg-negative may mediate inhibition of innate immunity. However, the current study is weakened by a lack of suitable control experiments. Thus, this model provides a control model of HBeAg-negative for studying the role of e antigen in HBV infection and provides a tool for the screening of antiviral drugs for HBeAg-negative HBV infection.

The major feature of our animal model was a viral template that originated from a patient. We found a nucleotide point mutation at residue 1896 that changed G to A (G1896A) in the preC region, and a double mutation (A1762T and G1764A) in the basal core promoter region of this DNA template. The preC G1896A mutation results in a stop codon that prematurely terminates the synthesis of HBeAg [27]. The basal core promoter region double mutation decreases HBeAg expression up to 70%, but enhances viral genome replication [27–29]. Since 1989, liver specialists have studied the mechanism of HBeAg-negative HBV infection from the perspective of virus variations [30,31]. HBV mutations are generated primarily due to a lack of proofreading capacity by HBV polymerase and because of host immune pressure. Several types of HBV preC/C mutations have been identified. Currently, the classic mutations include G1896A and the double mutation, A1762T/G1764A [6,12,32,33]. More than a decade of follow-up studies have revealed that patients in the inactive phase of hepatitis B infection, harboring genotype B viruses, are at high risk for reactivation [34]. Thus, using patient HBV DNA as a template could be representative of clinical infection. Moreover, compared to other models, the HBV DNA template root in the clinic is due to a lack of proofreading capacity during reverse transcription and a high replication rate; thus, HBV exists as a quasispecies [32]. This is advantageous to the virus for survival in adverse circumstances. We used such a clinical virus strain to establish a mouse model of HBeAg-negative HBV infection. This model will be valuable and reliable for evaluating the functional properties of clinical HBV isolates and for predicting their response to antiviral drugs.

cccDNA plays a key role in the life cycle of HBV and permits persistence of infection [35,36]. Currently, there are no antiviral drugs that can cure chronic HBV infection. Although nucleotide analogue antiviral drugs efficiently inhibit HBV replication, they fail to completely eliminate cccDNA. A virus rebound almost inevitably occurs soon after cessation of treatment as viral replication resumes using residual cccDNA as the template [37]. Therefore, it is necessary to express cccDNA persistently to mimic HBV infection [38,39].

To date, two simple methods have been described to produce recombinant cccDNA (rcccDNA), namely Cre-loxP-mediated site-specific DNA recombination [40] and minicircle technology [41,42]. These methods can produce large quantities of rcccDNA in a short time. However, to produce rcccDNA, these methods rely on the addition of an extra gene into the HBV genome. This could result in incomplete or imperfect HBV DNA. In contrast to those methods, we produced HBeAg-negative circularized DNA by a conventional technique based on plasmid-enzymatic ligation. This method is simple, convenient, and fast. Moreover, the circularized DNA contains no exogenous genes, thereby mimicking natural infection and representing a more reliable model for studying the HBeAg-negative HBV lifecycle and pathogenesis. In addition, this new model of persistent cccDNA expression can be used to screen new drugs and to evaluate therapeutic efficacy. Importantly, we have also successfully established a mouse model of HBeAg-positive HBV using this method [17].

In conclusion, an HBV-tolerant immunocompetent C57BL/6J mouse model that effectively simulates HBeAg-negative HBV infection was created successfully, for the first time, by a simple, rapid, and convenient method. It can be used for the evaluation of new drugs and for screening of antiviral drugs for clinical treatment of HBeAg-negative HBV infection. This mouse model also provides a tool to study the function of HBeAg as an immunogen and toleragen during HBV infection, along with its association with HBV persistence by using site-mutation to establish a comparable HBeAg-positive HBV model. Moreover, this model lays the foundation to establish individual HBV infection models using patient sera as template DNA. Drugs can then be evaluated using this model to achieve effective treatment.

## Supporting information

S1 Fig Generation of replication-competent HBV forms (ccc DNA) from patient DNA. Primers targeting the precore region permit amplification of the full-length HBV genome from virion-associated DNA. Full-length HBV was cloned into the pEASY-Blunt Simple Cloning vector. cccDNA was obtained by plasmid-enzymatic ligation.
(DOC)

S2 Fig Verification of cccDNA construction. Lane 1: 5000bp Marker; Lane 2: input linear DNA for ligation (full-length 3.2kb HBV genome after BspQI digestion and gel purefication); Lane 3: products after ligation; Lane 4: linear input DNA + EcoRI; Lane 5: ligated products + EcoRI; Lane 6: linear input DNA + PSAD; Lane 7: ligated products + PSAD; Lane 8: ligated products + PSAD + EcoRI.
(DOC)

S3 Fig The sensitivity of RCA+PCR. Lane 1: 600bp Marker; Lane 2: 103 copies; Lane 3: 102; lane 4:10 copies; Lane 5: 5 copies; Lane 6: 2.5 copies; Lane 7: 1 copy; Lane 8: negative control.
(DOC)

S1 File Supporting dataset. This file is used as a minimal data set.
(DOC)
