# Supplementary material for: A new model mimicking persistent HBV e antigen-negative infection using covalently closed circular DNA in immunocompetent mice
Source: PLoS One. 2017 Apr 20;12(4):e0175992. doi: 10.1371/journal.pone.0175992 (PMC5398701; doi:10.1371/journal.pone.0175992)
Supplement: S1 Fig — Primers targeting the precore region permit amplification of the full-length HBV genome from virion-associated DNA. Full-length HBV was cloned into the pEASY-Blunt Simple Cloning vector. cccDNA was obtained by plasmid-enzymatic ligation. (DOC) [file pone.0175992.s001.doc]

**Supporting information of figure**


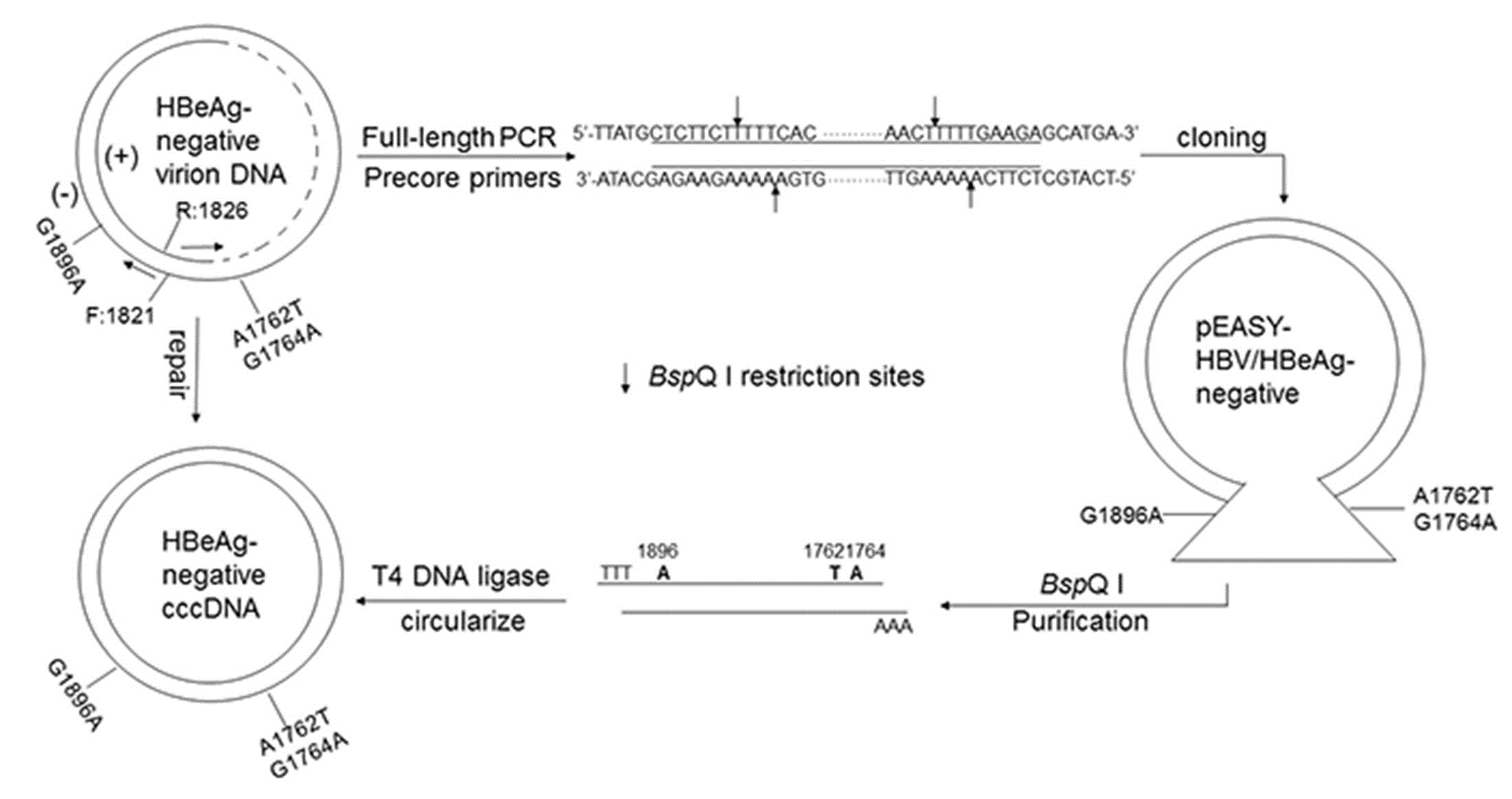


**S1 Fig.** **Generation of replication-competent HBV forms (ccc DNA) from patient DNA.** Primers targeting the precore region permit amplification of the full-length HBV genome from virion-associated DNA. Full-length HBV was cloned into the pEASY-Blunt Simple Cloning vector. cccDNA was obtained by plasmid-enzymatic ligation.
