# Supplementary material for: A new model mimicking persistent HBV e antigen-negative infection using covalently closed circular DNA in immunocompetent mice
Source: PLoS One. 2017 Apr 20;12(4):e0175992. doi: 10.1371/journal.pone.0175992 (PMC5398701; doi:10.1371/journal.pone.0175992)
Supplement: S2 Fig — Lane 1: 5000bp Marker; Lane 2: input linear DNA for ligation (full-length 3.2kb HBV genome after BspQI digestion and gel purefication); Lane 3: products after ligation; Lane 4: linear input DNA + EcoRI; Lane 5: ligated products + EcoRI; Lane 6: linear input DNA + PSAD; Lane 7: ligated products + PSAD; Lane 8: ligated products + PSAD + EcoRI. (DOC) [file pone.0175992.s002.doc]

**Supporting information of figure**


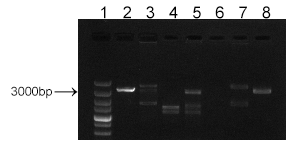


**S2 Fig.** **Verification of cccDNA construction**. Lane 1: 5000bp Marker; Lane 2: input linear DNA for ligation (full-length 3.2kb HBV genome after BspQI digestion and gel purefication); Lane 3: products after ligation; Lane 4: linear input DNA + EcoRI; Lane 5: ligated products + EcoRI; Lane 6: linear input DNA + PSAD; Lane 7: ligated products + PSAD; Lane 8: ligated products + PSAD + EcoRI.
