# Supplementary material for: A new model mimicking persistent HBV e antigen-negative infection using covalently closed circular DNA in immunocompetent mice
Source: PLoS One. 2017 Apr 20;12(4):e0175992. doi: 10.1371/journal.pone.0175992 (PMC5398701; doi:10.1371/journal.pone.0175992)
Supplement: S3 Fig — Lane 1: 600bp Marker; Lane 2: 103 copies; Lane 3: 102; lane 4:10 copies; Lane 5: 5 copies; Lane 6: 2.5 copies; Lane 7: 1 copy; Lane 8: negative control. (DOC) [file pone.0175992.s003.doc]

**Supporting information of figure**


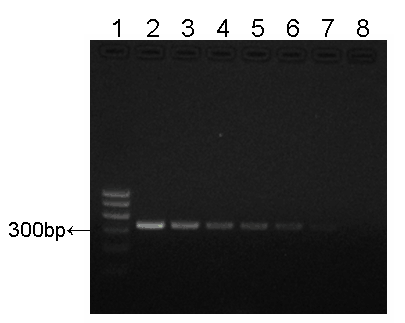


**S3 Fig. The sensitivity of RCA+PCR.** Lane 1: 600bp Marker; Lane 2: 10^3^ copies; Lane 3: 10^2^; lane 4:10 copies; Lane 5: 5 copies; Lane 6: 2.5 copies; Lane 7: 1 copy; Lane 8: negative control.
