# Supplementary material for: A new model mimicking persistent HBV e antigen-negative infection using covalently closed circular DNA in immunocompetent mice
Source: PLoS One. 2017 Apr 20;12(4):e0175992. doi: 10.1371/journal.pone.0175992 (PMC5398701; doi:10.1371/journal.pone.0175992)
Supplement: S1 File — This file is used as a minimal data set. (DOC) [file pone.0175992.s004.doc]

**Supporting dataset**

**S1 Table.** **Levels of HBsAg in sera after hydrodynamic injection (ng/ml)**

**S2 Table.** **Levels of HBeAg in sera after hydrodynamic injection (nuc/ml)**

**S3Table. Levels of HBV DNA in sera (10^6^copies /ml)**

**S4 Table. Levels of HBV DNA in liver tissue (10^7^copies /ml)**

**S File. HBV gene sequence**

tttttcacctctgcctaatcatctcttgttcatgtcctactgttcaagcctccaagctgtgccttgggtggctttaggacatggacattgacccgtataaagaatttggagcttcagtggagttactctcttttttgccttctgacttctttccttctattcgagatctcctcgacaccgccgctgctctgtatcgggaggccttagagtctccggaacattgtacacctcaccatacggcactcaggcaagctattctgtgttggggtgagttaatgaatctagccacctgggtgggaagcaatttggaagatccagcatccagggaagcagtagtcagctatgtcaacgttaatatgggcctaaaaattagacaactattgtggtttcacatttcctgtcttacttttgggagagacactgttcttgaatatttggtgtcttttggagtgtggattcgcactcctcctgcatatagaccacaaaatgcccctatcttatcaacacttccggaaactactgttgttagacaaagacgcaggtcccctagaagaagaactccctcgcctcgcagacgaaggtctcaatcgccgcgtcgcagaagatctcaatctcgggaacgtcaatgttagtattccttggacacataaggtgggaaactttacggggctttattcttctacggtaccttgctttaatcctaaatggcaaactccttcttttcctgatattcatttgcaggaggacattgttgatagatgtaagcaatttgtggggccccttacagtaaatgaaaacaggaaactaaaattaattatgcctgctaggttttatcccaaggccactaaatatttgcccttagataaagggatcaaaccgtattatccagagcatgtagttaatcattacttccagacgcgacattatttacacactctttggaaggcggggatcttatataaaagagagtccactcgtagcgcctcattttgcgggtcaccatattcttgggaacaagatctacagcatgggaggttggtcttccaaacctcgaaaaggcatggggacaaatctttctgtccccaatcccctgggattcttccccgatcatcagttggaccctgcattcaaggccaactcagaaaatccagattgggacctcaacccgcacaaggacaactggccggacgccaaccaggtgggagtgggagcattcgggccagggttcactcctccccatgggggaatgatggggtggagccctcaggcccagggcctactcacagctgtgccagcagctcctcctcctgcctccaccaatcggcagtcaggaaggcagcctactcccttatctccacctctaagggacactcatcctcaggccatgcagtggaattccaccactttccaccaaactcttcaagatcccagagtcagggccctgtaccttcctgctggtggctccagttcaggaacattgaaccctgctcagaatactgtctctgccatatcgtcaatcttatcaacgactggggaccctgtaccgaacatggagaacatcgcatcaggactcctaggacccctgctcgtgttacaggcggggtttttcttgttgacaaaaatcctcacaataccacagagtctagactcgtggtggacttctctcaattttccagaggggacacccgtgtgtcttggccaaaattcgcagtcccaaatctccagtcactcaccaacctgttgtcctccaatttgtcctggttatcgctggatgtgtctgcggcgttttatcatcttcctctgcatcctgctgctatgcctcatcttcttgttggttcttctggactatcaaggtatgttgcccgtttgtcctctaattccaggatcatcaacaaccagcacgggaccatgcaaaacctgcacgactcctgctcaaggaacctctatgtttccctcatgttgctgtacaaaacctacggacggaaactgcacctgtattcccatcccatcatcttgggctttcgcaaaatacctatgggagtgggcctcagtccgtttctcttggctcagtttactagtgccatttgttcagtggttcgtagggctttcccccactgtctggctttcagttatatggatgatgtggttttgggggccaagtctgtacaacatcttgagtccatttataccgctgttaccaattttcttttgtctttgggtatacatttaaaccctcacaaaactaaaagatggggatattcccttaacttcatgggatatgtaattgggagttggggcacattgccacaggaacatattgtacaaaaaatcaaaatgtgttttaggaaacttcctgtaaacaggcctattgattggaaagtatgtcaacgaattgtgggtcttttgggatttgccgctcctttcacacagtgtggatatcctgctttaatgcctttatatgcatgtatacaagcaaaacaggcttttactttctcgccaacttacaa

ggcctttttaagtaaacagtatctgaacctttaccccgttgctcggcaacggcctggtctgtgccaagtgtttgctgacgcaacccccactggttggggcttggccatcggccatcagcgcatgcgtgggacctttgtgtctcctctgccgatccatactgcggaactcctagccgcgtgttttgctcgcagccggtctggggcaaaactcatcgggactgacaattctgtcgtgctctcccgcaagtatacatcatttccatggctgctaggctgtgctgccaactggatcctgcgcgggacgtcctttgtttacgtcccgtcggcgctgaatcccgcggacgacccctcccggggccgcttggggctctaccgcccgcttctccgcctgtcgtaccgaccgaccacggggcgcacctctctttacgcggactccccgtctgtgccttctcatctgccggaccgtgtgcacttcgcttcacctctgcacgtcgcatggagaccaccgtgaacgcccacagaaacctgcccaaggtcttgcataagaggactcttggactttcagcaatgtcaacgaccgaccttgaggcatactttaaagactgtgtgtttgctgagtgggaggagttgggggaggaggttaggttaatgatctttgtactaggaggctgtaggcataaattggtgcgttcaccagcaccatgcaac
